# Effect of polyunsaturated fatty acids on proliferation and survival of prostate cancer cells

**DOI:** 10.1371/journal.pone.0219822

**Published:** 2019-07-17

**Authors:** Brenden A. Bratton, Ivan V. Maly, Wilma A. Hofmann

**Affiliations:** Department of Physiology and Biophysics, Jacobs School of Medicine and Biomedical Sciences, University at Buffalo, Buffalo, NY, United States of America; Laval University, CANADA

## Abstract

Progression of prostate cancer to lethal forms is marked by emergence of hormone-independent proliferation of the cancer cells. Nutritional and epidemiological studies have indicated that prostate cancer progression is correlated with the consumption of polyunsaturated fatty acids (PUFA). To shed additional light on the cell-level mechanisms of the observed correlation, we compared the sensitivity of hormone-dependent and hormone-independent prostate cancer cells to growth medium supplementation with free PUFAs in a cell proliferation and viability assay. Our data show that the hormone-dependent cells are comparatively insensitive to various PUFAs, at the same time as the growth and viability of hormone-independent cells lines are strongly inhibited by most of the tested PUFAs, whether *n*–3 or *n*–6. We speculate that this difference may be at least partially responsible for the observed effects of specific dietary lipids in prostate cancer. The new data strengthen the case for dietary intervention as part of potential new therapeutic strategies seeking to impede prostate cancer progression.

## Introduction

Prostate cancer is one of the most common malignancies; over 3 million men are living with this condition in the United States alone [1, 2]. The numbers reflect this cancer’s relatively long period of indolence, but prognosis and treatment remain difficult in view of the transition to the disseminated and lethal stages [3]. The difficult-to-manage stages are often separated from the period believed to merit only watchful waiting by the emergence of castration resistance [4]. Epidemiological research has established a correlation of not only the disease incidence, but also its progression with nutrition, particularly with the intake and metabolism of fatty acids [5–8].

For example, Mediterranean diet was found to be associated inversely with prostate cancer incidence [9]. The positive effect of the Mediterranean diet was qualitatively attributable, in part, to the high content of *n*–3 fatty acids from fish. The issue remains complicated, however, as other work has shown a lack of association of the post-diagnosis outcome with the adherence to Mediterranean diet [10]. The traditional Argentinian diet, distinguished by the high red-meat content, has also been compared with the alternative diets in the same population and found to be associated with the risk of prostate cancer [11]. Notably, meat in the Argentinian (“Southern Cone”) diet is a major source of fat [12], contributing primarily saturated fatty acids and C18:2 *n*–6 linoleic acid (LA). While the intake of individual *n*–3 and *n*–6 PUFAs was not correlated with prostate cancer risk in a case-control study on a United States cohort [13], the *n*–6 to *n*–3 ratio in the individual’s diet was significantly correlated with the risk of high-grade prostate cancer. Since it lacked correlation with the low-grade disease, a conclusion could be drawn that the disease progression was specifically impacted by the dietary choices.

Experiments on the prostate-specific *Pten* knockout mouse model have been in agreement with the above conclusion, showing an induction of Bad-dependent apoptosis in the tumors and delayed progression on *n–*3 rich diet, while *n*–6 rich diet had the opposite effect [14]. Work on nude mice injected with human prostate cancer cells DU145 [15], at the same time, showed that while diet rich in EPA and DHA (C20 and C22 *n*–3) led to smaller tumors compared with diet rich in LA (C18 *n*–6), tumors in animals fed with a diet rich in ALA (C18 *n*–3) were similar to the latter group. A similar difference in effectiveness between the long-chain and short-chain *n*–3 PUFAs was seen in other studies utilizing this mouse model [16–18]. Explanation of the complexity and contradiction in the epidemiological and animal work may benefit from information that could be obtained in experiments in vitro. The picture arising from in vitro experiments, however, is also far from complete. In a system combining artificial matrigel basement membrane barrier and bone marrow stroma as the attractant, evidence was obtained for enhancement of prostate cancer cell migration by the C20:4 *n*–6 arachidonic acid (AA) [19]. This effect is mediated by the AA conversion into, in particular, prostaglandin E2, and it is reversed by *n*–3 PUFA eicosapentaenoic and docosapentaenoic acids (EPA, DHA), apparently via a competitive inhibition mechanism. The reversing action demonstrated in this experiment is similar to the *n*–3 sensitivity of the earlier-reported proliferative and anti-apoptotic action of AA [17, 18, 20]. The proliferative action of AA was observed between 0.5–40 μM, using complexation with fatty-acid-free bovine serum albumin (BSA), on hormone-independent PC3 cells under the conditions of supplementation with 0.5% FBS, and similar results were obtained on hormone-dependent LNCaP cells [21]. This effect was found to be mediated by the downstream 5-lypoxygenase metabolite 5-hydroxyeicosatetraenoic acid, while inhibition of 5-lypooxygenase triggered apoptosis [22].

More recently, Meng et al. [23] studied the fatty acid composition of prostate cells in vitro and determined that malignant prostate cancer cells (PC-3) had significantly less *n*–3 PUFA– α-linolenic acid (ALA), EPA, and DHA–than the normal prostate epithelial cells (RWPE-1), but notably the content of *n*–6 LA was also less. Supplementation of the culture medium with these fatty acids, and also with γ-linolenic acid (GLA) and AA, resulted in increase of the cellular content of the corresponding fatty acid and its long-chain derivatives. There were differences in the response between the normal and malignant cells, and evidence for retroconversion as well as cross-talk between the *n*–3 and *n*–6 pathways in the supplementation experiments. Production of TNF-α, IL-6, LXA_4_, and free radicals was impacted, and the cells’ growth and viability, as measured by 3-dimethylthiazolyl-2,5-diphenyltetrazolium (MTT) colorimetric assay for mitochondrial activity, were strongly suppressed. In the fatty acid concentration range tested (5–240 μM), evidence was found for a higher sensitivity of growth and viability of the normal cells relative to the metastatic ones.

This line of work was extended in the most recent experiments [24] that were conducted on another hormone-independent prostate cancer cell line, DU145 [25]. BSA was employed to solubilize fatty acids and model the physiological role of serum and interstitial albumin in transporting and presenting fatty acids to cells [26–29]. In the MTT assay, BSA alone did not impact the cell viability, but treatment with BSA-conjugated DHA reduced it significantly. EPA had a smaller effect, and AA (tested in the range 10–100 μM for 24 h) had no significant influence. The further studied DHA effects included characteristic apoptotic nuclear condensation and segmentation, and apoptosis-related gene expression changes. The apoptosis inhibitor XIAP was found to be downregulated, while caspases 1, 3, and 9, TP53, and tumor necrosis-related factors TNF and TNFRSF1A were upregulated more than 2-fold.

The cited results establish sensitivity of the viability of hormone-independent prostate cancer cells to exposure to PUFA (*n*–3 and, generally, also *n*–6) and provide evidence for a cell-level, apoptosis-linked mechanism that may play a role in the effects noted in the epidemiological and nutritional studies. At the same time, one new question raised by the cited work concerns the possibility of a difference between the sensitivity of cells representing different stages of prostate cancer progression, such as those that are hormone-dependent and hormone-independent. Any evidence–pro or contra–might have a bearing on the cell-level mechanisms behind the role of PUFA specifically in prostate cancer progression. An in vitro analysis aimed at addressing this question is undertaken in the present work. In addition to PC3 cells, we tested the hormone-dependent LNCaP line and its hormone-independent derivative C4-2 [25, 30, 31]. FFA concentrations in arterial blood depend on the physiological condition, falling within the range 100–1000 μM [26]. LA accounts for approximately 10% of this level. Thus, we were interested in testing the cells’ response to PUFA in a range bracketing 10–100 μM, as in the cited recent in vitro work. BSA conjugation was employed for the presentation of a panel of *n*–3 and *n*–6 PUFA, and cell viability was assessed using the MTT assay. The new findings indicate that advanced, hormone-independent prostate cancer cells may be more sensitive to the cytotoxic action of PUFA, supporting a role for *n*–3 and *n*–6 PUFA in prostate cancer progression.

## Materials and methods

### Chemicals and reagents

MTT bromide suitable for cell culture was purchased from Sigma-Aldrich (St. Louis, MO). Fatty-acid-free BSA (catalog number A7030) and the following fatty acids suitable for cell culture and ≥99% pure were purchased from Sigma-Aldrich (St. Louis, MO): LA, GLA, AA, ALA, EPA (*cis*-5,8,11,14,17), DHA (*cis*-4,7,10,13,16,19).

### Preparation of fatty acids

Fatty acids used in this study were complexed with fatty-acid-free BSA at a 5:1 molar ratio. Fatty-acid-free BSA was reconstituted in 150 mM NaCl to obtain a 2 mM BSA solution. Fatty acids were then added to obtain a 10 mM fatty acid, 2 mM BSA stock solution. The solution was incubated for 2 h at 37°C with vigorous shaking to encourage coupling of fatty acids to BSA. The stock solution was kept at –20°C. Fatty acids and prepared stock solutions were kept at all times under argon to prevent oxidation. For treatment of cells, the stock solution was diluted in serum-free RPMI 1640 medium to obtain the various treatment concentrations.

### Cell culture and fatty acid treatment

PC-3, LNCaP, and C4-2 cells were purchased from ATCC (American Type Culture Collection, Manassas, VA) and routinely cultured in RPMI 1640 medium without phenol red (Invitrogen, Calrsbad, CA) supplemented with 10% fetal bovine serum and 1% penicillin/streptomycin, at 37°C with 5% CO2. (Cell line references, ATCC catalog numbers, and purchase dates: PC-3 [32]–CRL-1435, 01/2014; LNCaP [33]–CRL-1740, 01/2014; C4-2 [34]–CRL-3314, 01/2014. The cells were acquired from the repository directly.)

For fatty acid treatment, cells (1·10^4^ per well) were seeded in 96-well plates and incubated for 24 h in 100 μL of culture medium. Before treatment with fatty acids, the culture medium was removed and the cells were equilibrated for 2 h in serum-free RPMI 1640 medium. The equilibration medium was then replaced by serum-free RPMI 1640 medium enriched with BSA-coupled fatty acids at various concentrations for 24 h, 48 h, or 72 h. Some of these time courses had the same start time (different length of treatment being applied to the same cell passage), and some had different start times (treatment applied to different cell culture passages). Negative control cells received medium with fatty-acid-free BSA at equal concentrations.

### Cell proliferation and survival assay

Cell proliferation and survival was assessed by the MTT colorimetric method [35]. MTT was reconstituted in PBS to obtain a stock solution of 5 mg/ml. After cell treatment (described above), the culture medium was removed from the wells and replaced with an equal volume of MTT treatment solution. The MTT treatment solution was prepared by adding 10% (by volume) of the MTT stock solution to RPMI 1640 without phenol red. The cells were incubated with the MTT treatment solution for 1 h at 37°C under 5% CO_2_. The solution was then removed, and 100 μL DMSO were added to each well to solubilize the MTT formazan crystals. The plate was incubated for 20 min on an orbital shaker with agitation, and the absorbance was measured at 540 nm using a BioTek Synergy microplate reader (BioTek Instruments, Inc., Winooski, VA). The cell viability was calculated as 100% × (absorbance of treated wells–absorbance of blank control wells) / (absorbance of negative control wells–absorbance of blank control wells). The blank control wells were treated identically to the other wells, except that no cells were seeded and the medium was free of BSA and fatty acids. The MTT experiments were performed in triplicate (on three cell culture wells) and, as a rule, additionally repeated on different cell passages.

### Statistical analysis

No results were excluded from the analysis. Statistical significance of the deviation of the mean from the negative control level was assessed by the confidence interval method. A conservative approach was taken by applying the Dunn-Bonferroni correction for multiple comparisons, corresponding to the total error rate (significance level) α = 0.05 for the entire set of experimental conditions in this paper. There are *m* = 324 experimental conditions (cell lines × fatty acids × time points × concentrations). Accordingly, the confidence intervals for each data point were calculated based on the significance level α/*m* = 1.5 10^−4^.

## Results

### Effects of AA (C20 *n*–6)

**Fig 1:**
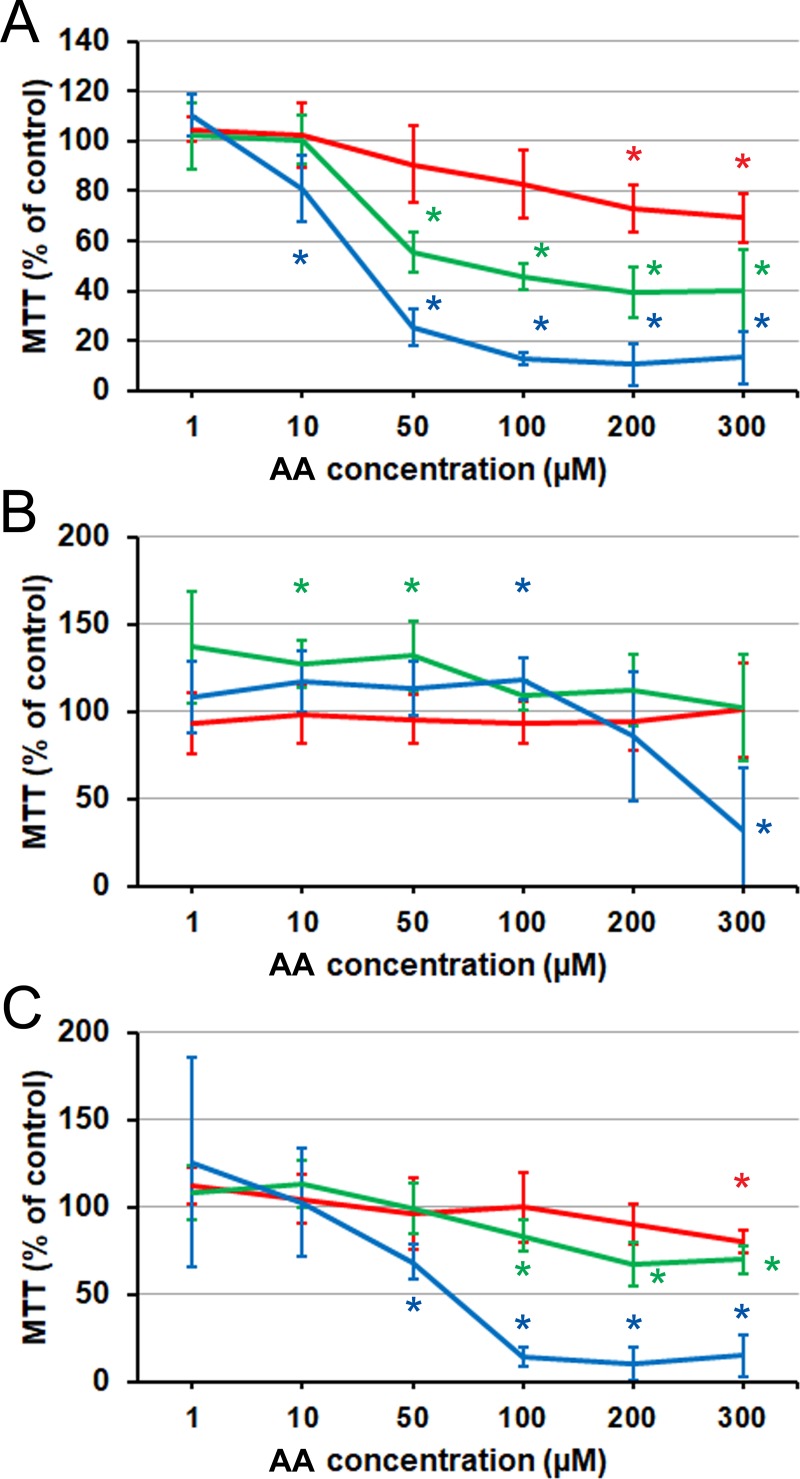
Effect of AA on prostate cancer cells proliferation and viability as measured by MTT assay, relative to the BSA carrier control. (A) PC3 cell line. (B) LNCaP cell line. (C) C4-2 cell line. Red line: 24 h. Green line: 48 h. Blue line: 72 h. Error bars: standard deviation. Asterisks denote statistical significance of the difference from the control on the significance level 0.05, corrected for multiple comparisons (see Methods). 24 h data: each point is the average of *n* = 6 cell cultures. 48 and 72 h data: each point is the average of *n* = 9 cell cultures.

As expected, exposure of PC3 cells to AA resulted in suppression of the cell viability measured by MTT assay (Fig 1A). The inhibition was notably progressive, with the numbers suggesting that few viable cells remained after 72 h, when concentrations above 50 μM were used. Having confirmed that the behavior of this hormone-independent line was essentially as in the cited earlier experiments, we proceeded to test the action of AA on the hormone-dependent prostate cell line LNCaP. After 24 h of exposure, no effect was observed (Fig 1B). At 48 h, a range of intermediate concentrations (10–50 μM) was mildly stimulatory. After 72 h, 100 μM was mildly stimulatory while the very high (300 μM) concentration was strongly inhibitory. Tentatively, the difference from the PC3 response was compatible with the hypothesis that the response to PUFA may be dependent on the stage of prostate cancer. To test this further, we examined the action of AA on the hormone-independent derivative of LNCaP, C4-2. The response (Fig 1C) was essentially identical to PC3, but with a more marked time-dependence.

### Long-chain *n*–3 PUFAs

**Fig 2:**
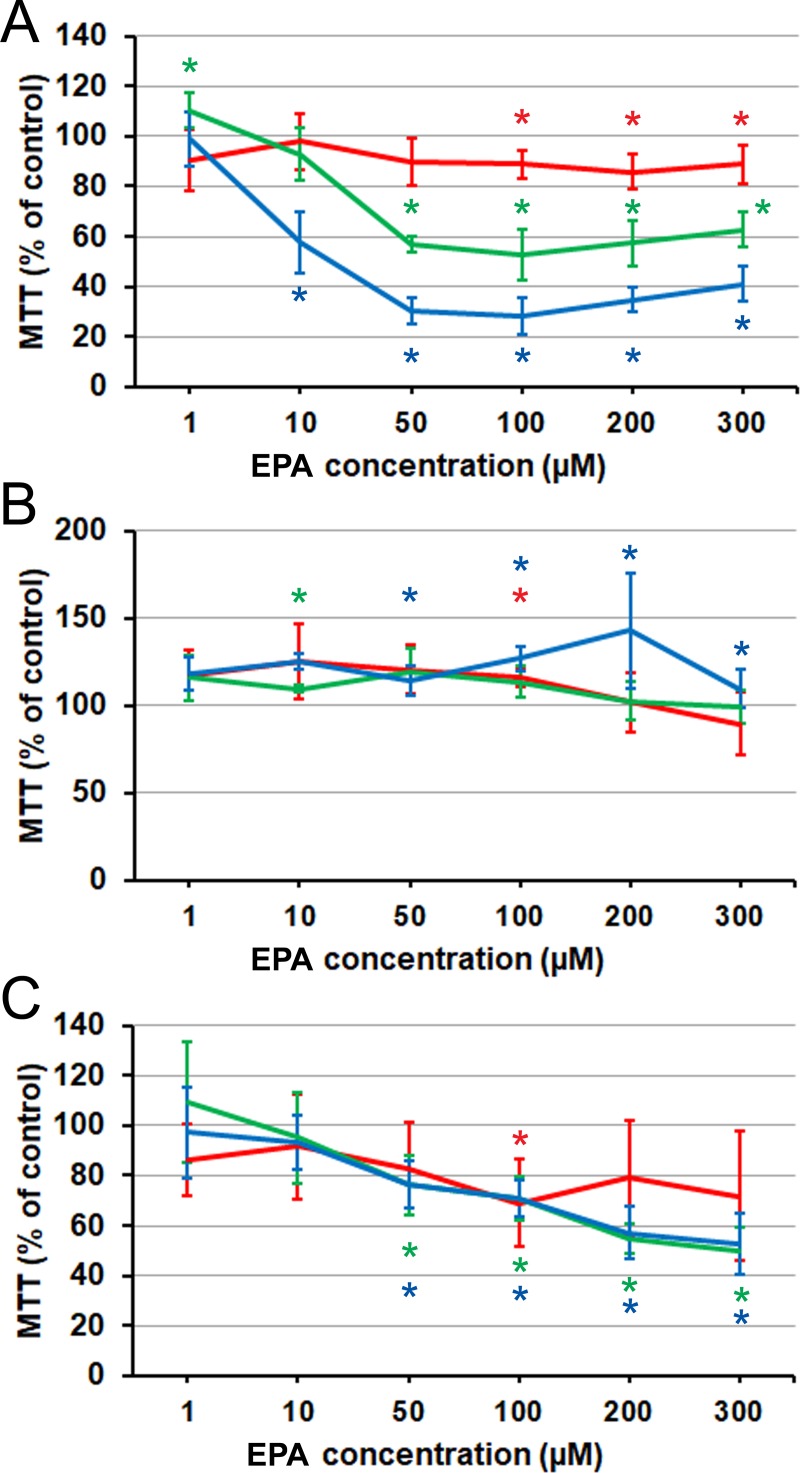
Effect of EPA on prostate cancer cells proliferation and viability as measured by MTT assay. (A) PC3 cells. (B) LNCaP cells. (C) C4-2 cells. Red line: 24 h. Green line: 48 h. Blue line: 72 h. Error bars: standard deviation. Asterisk: difference from the control is significant on the significance level 0.05, corrected for multiple comparisons. PC3 and C2-4 data: each point is the average of *n* = 9 cell cultures. LNCaP data: each point is the average of *n* = 6 cell cultures.

We next tested whether long-chain *n*–3 PUFAs acted similarly or in the opposite sense. EPA (C20:5) acted on PC3 cells similarly to AA, resulting generally in a strong inhibition (Fig 2A). Two small additional effects could be noted. Firstly, the concentration dependence was slightly biphasic, with the highest concentrations appearing somewhat less effective than the intermediate ones. Secondly, the lowest concentration (1 μM) caused a small, transient stimulation of the cell growth.

LNCaP cells exposed to EPA did not show any inhibition but, instead, a degree of stimulation (Fig 2B). Comparing this observation with the data obtained on this cell line with AA, the stimulatory effect of EPA was similar in magnitude but not restricted to the lower concentrations. C4-2 cells responded to EPA with a comparatively moderate but rapidly developing inhibition (Fig 2C). Overall, comparing EPA with AA, the pattern of response of the hormone-independent cell lines (inhibition) was common to both *n*–3 and *n*–6 C20 PUFAs, while the difference with the hormone-dependent cell line was even more clear-cut with EPA than with AA.

**Fig 3:**
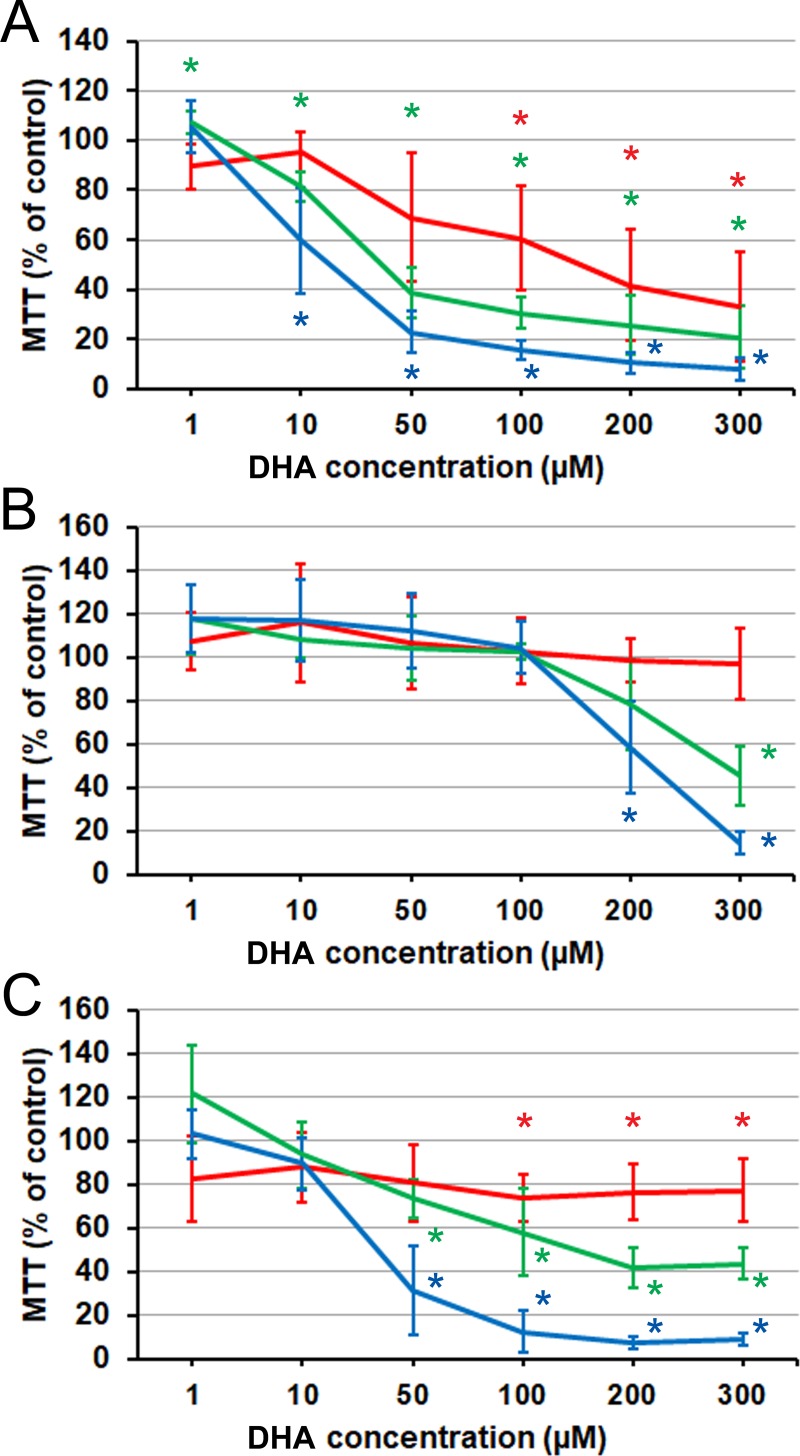
Effect of DHA on prostate cancer cells proliferation and viability as measured by MTT assay. (A) PC3 cells. (B) LNCaP cells. (C) C4-2 cells. Red line: 24 h. Green line: 48 h. Blue line: 72 h. Error bars: standard deviation. Asterisk: difference from the control is significant on the significance level 0.05, corrected for multiple comparisons. Each data point is the average of *n* = 9 cell cultures.

The pattern of action of DHA (C22:6) on the three cell lines (Fig 3) matched that of AA, except, primarily, that the stimulatory influence on LNCaP cells was completely absent. Inhibition of this cell line could be obtained earlier (from 48 h) than with AA at the highest concentration (300 μM), and by 72 h it could be obtained at a somewhat lower concentration (200 μM). As was also the case in the EPA experiments, a degree of stimulation was seen transiently (at 48 h) at the lowest concentration (1 μM) in PC3 cells. The suppression of C4-2 cells developed earlier at the intermediate concentrations, compared with AA, but the level reached by 72 h was as deep as with that PUFA, and markedly deeper than with EPA.

Overall, it can be said that the pattern of response of the tested cell lines to DHA was similar to the other long-chain PUFAs, whether *n*–3 or *n*–6. The hormone-responsive cell line was insensitive to their inhibitory action up to the highest concentrations (200–300 μM AA or DHA), while the two hormone-independent cell lines were sensitive to the inhibition starting from 10 μM (PC3) or 50 μM (C4-2) after prolonged exposure (72 h). EPA was remarkable for not being able to cause any inhibition of LNCaP cells in the range of conditions tested, while eliciting a moderate but consistent stimulation of this cell line through a range of concentrations.

### Short-chain precursors and essential fatty acids

**Fig 4:**
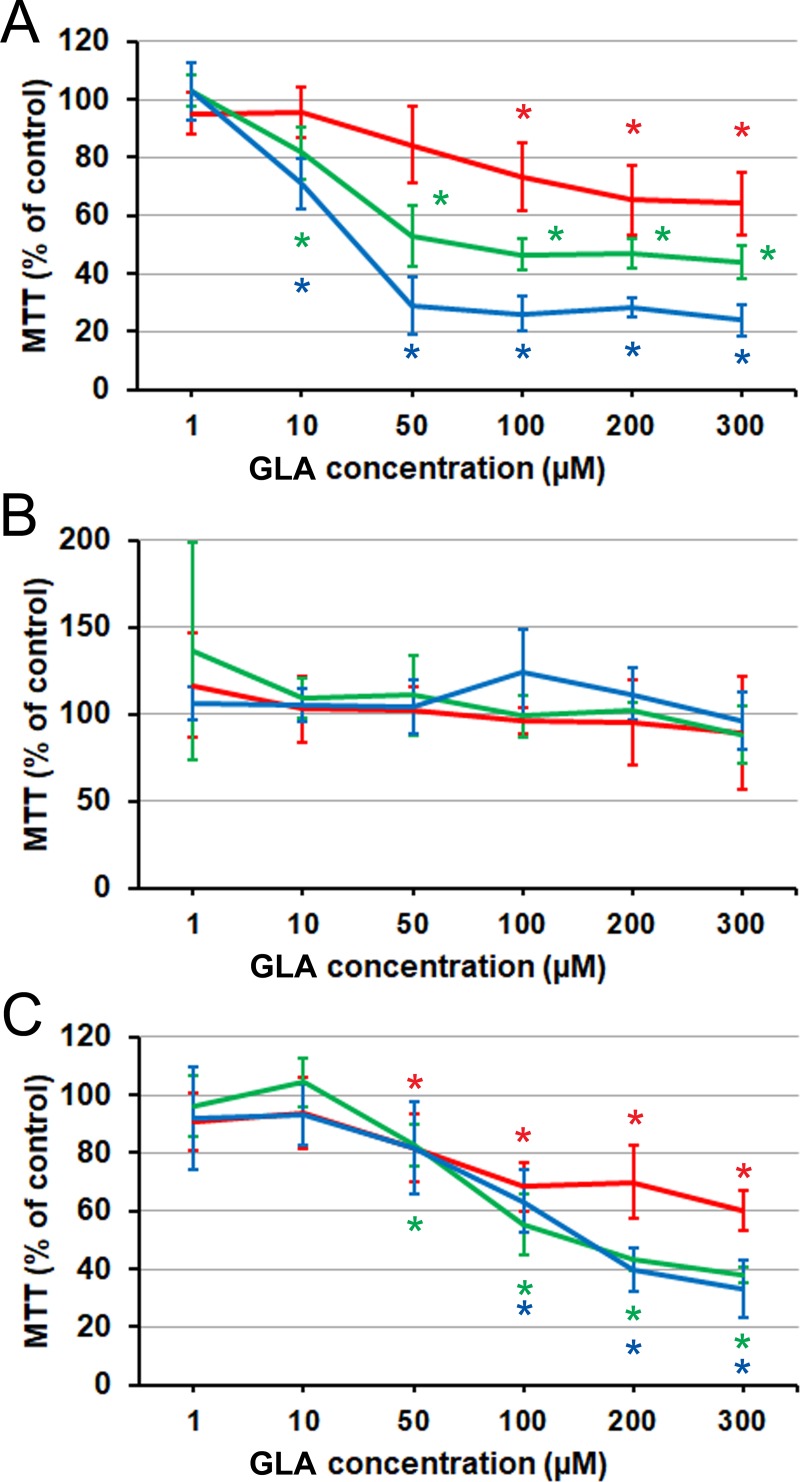
Effect of GLA on prostate cancer cells proliferation and viability as measured by MTT assay. (A) PC3 cells. (B) LNCaP cells. (C) C4-2 cells. Red line: 24 h. Green line: 48 h. Blue line: 72 h. Error bars: standard deviation. Asterisk: difference from the control is significant on the significance level 0.05, corrected for multiple comparisons. Each data point is the average of *n* = 9 cell cultures.

**Fig 5:**
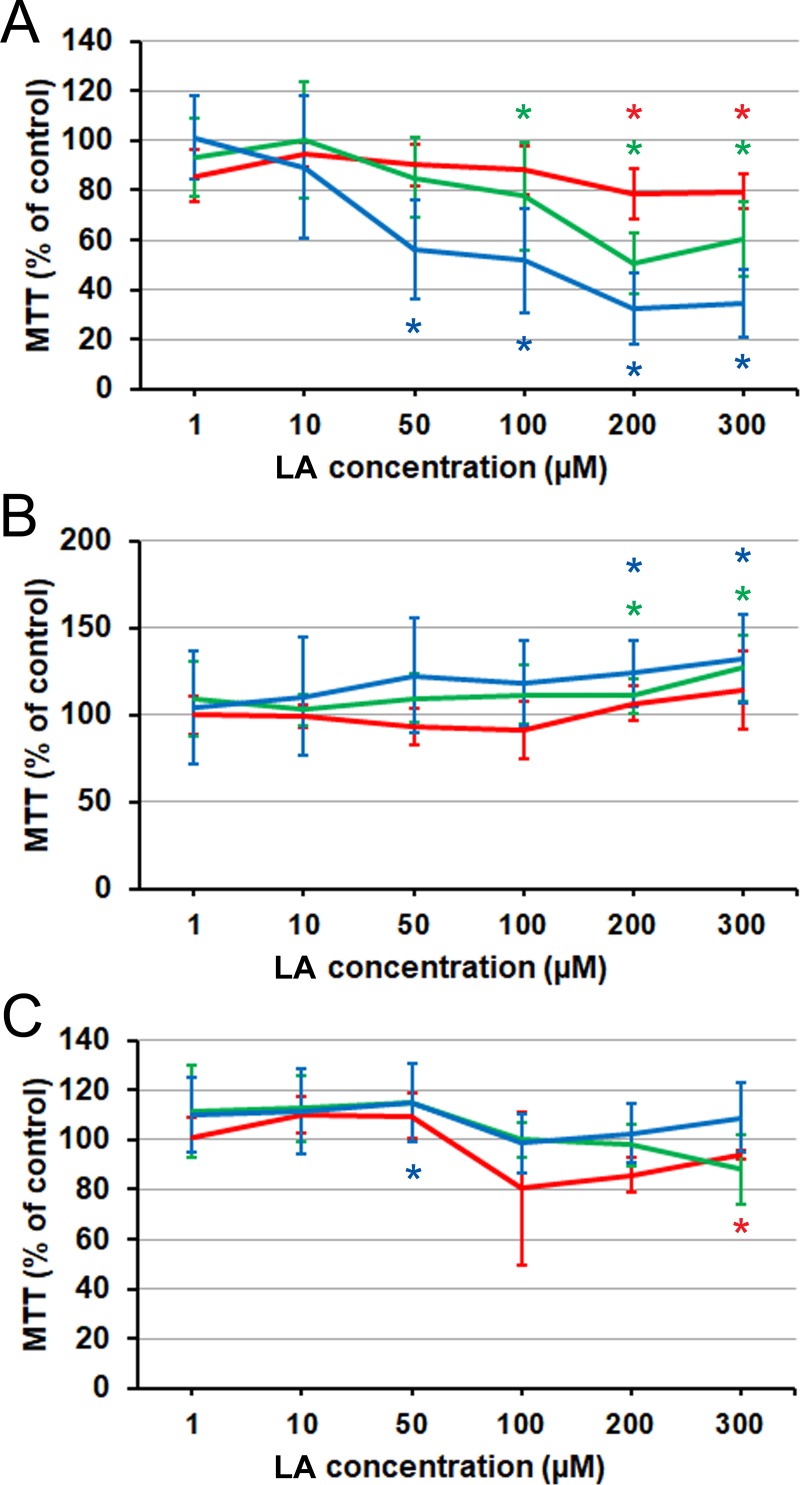
Effect of LA on prostate cancer cells proliferation and viability as measured by MTT assay. (A) PC3 cells. (B) LNCaP cells. (C) C4-2 cells. Red line: 24 h. Green line: 48 h. Blue line: 72 h. Error bars: standard deviation. Asterisk: difference from the control is significant on the significance level 0.05, corrected for multiple comparisons. C4-2 24 h data: each point is the average of *n* = 3 cell cultures. All other data: each point is the average of *n* = 9–21 cell cultures.

Next we examined whether supplementation with C18 precursors of AA [6] had any differential effect across the cell lines. GLA (18:3) elicited responses (Fig 4) that were similar to those of AA, except that neither stimulation nor inhibition was seen at any concentration in LNCaP cells. Responses to 18:2 LA differed, however, in that LNCaP showed only a stimulation at high concentration and C4-2 no longer had any definitive pattern of response (Fig 5). The transient effect in C4-2 at high concentration and the slowly developing stimulation at intermediate concentrations (Fig 5C) signified that the progressive inhibitory response elicited in these cells by AA is not mimicked, on this time scale, by its essential precursor. In LNCaP, at the same time, the elicited pattern can be seen as resulting from a delayed conversion into AA, as the high-concentration and late time-point stimulatory response to LA could correspond to the stimulation seen at intermediate concentrations with AA.

**Fig 6:**
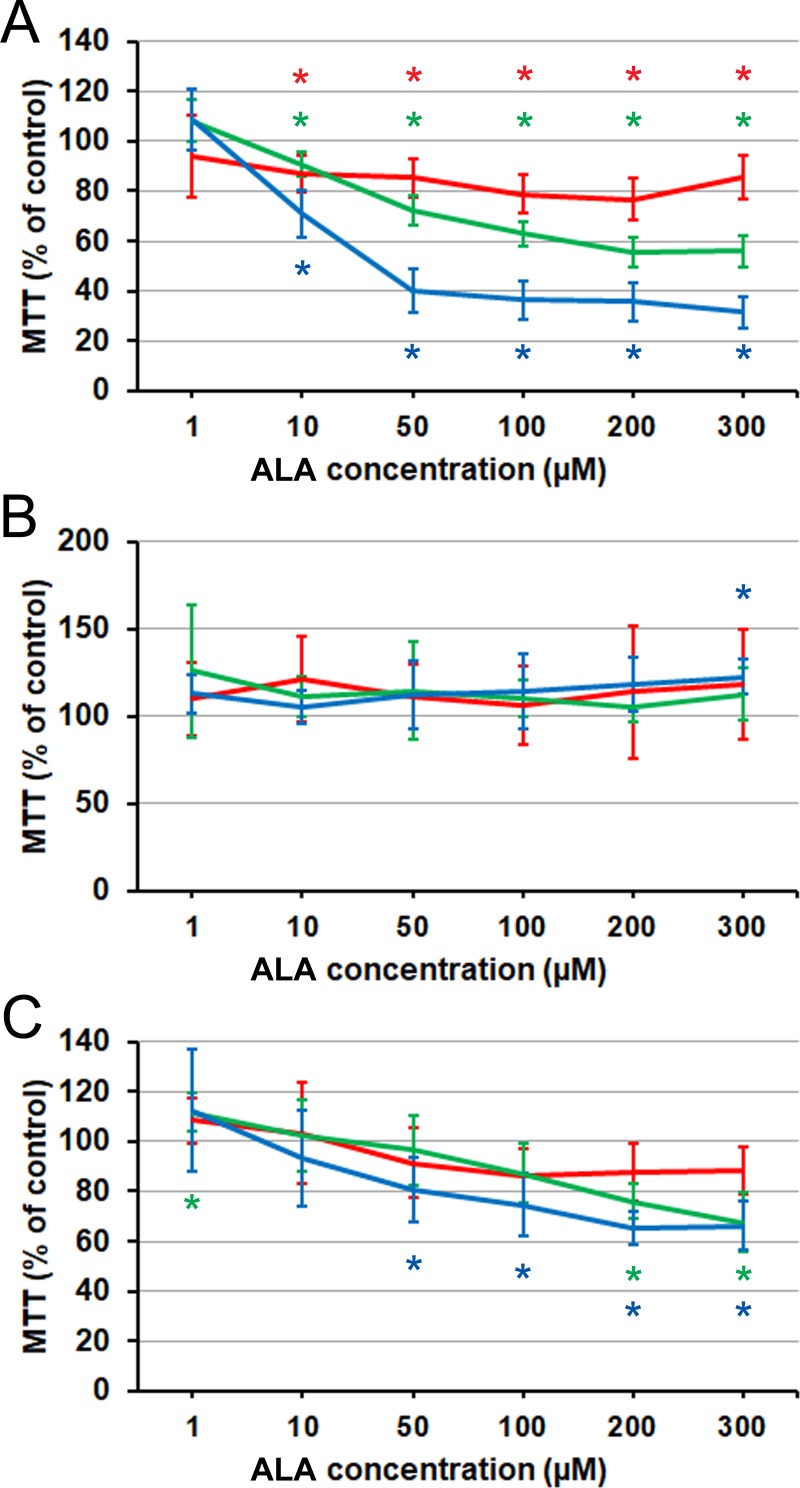
Effect of ALA on prostate cancer cells proliferation and viability as measured by MTT assay. (A) PC3 cells. (B) LNCaP cells. (C) C4-2 cells. Red line: 24 h. Green line: 48 h. Blue line: 72 h. Error bars: standard deviation. Asterisk: difference from the control is significant on the significance level 0.05, corrected for multiple comparisons. Each data point is the average of *n* = 9 cell cultures.

Finally, we assessed the cell lines’ response to the essential EPA and DHA precursor ALA (18:3 *n*–3). Identically to EPA and DHA, ALA induced a marked and progressive inhibition of PC3 cells at most concentrations, but the slight transient activation of their growth at the lowest concentration was no longer seen (Fig 6A). The effect of this PUFA on LNCaP cells–a mild stimulation of growth at the highest concentration and longest time (Fig 6B)–could be viewed as compatible with its slow conversion into EPA, which showed the stimulation earlier and at lower concentrations. The cells did not exhibit any of the high-concentration induced growth inhibition, which was characteristic of DHA, ALA’s more distant derivative. The response of C4-2 cells (Fig 6C) was qualitatively similar to both the EPA and DHA picture–inhibition at 50 μM and up–but was quantitatively milder than even that of EPA. An additional difference was a slight but statistically significant stimulation of growth at the lowest concentration of ALA (1 μM).

## Discussion

The dynamic picture arising from our new experiments can be summarized as follows. Both *n*–3 (ALA, EPA, DHA) and *n*–6 (LA, GLA, AA) PUFAs induced a strong and progressive suppression of the numbers of viable cells in the case of the metastatic, hormone-independent cell line PC3. A mild transient activation of these cells at the lowest concentration (1 μM) was also detected, with the long-chain *n*–3 PUFAs EPA and DHA. With the exception of DHA and GLA, the tested PUFAs were capable of stimulating the growth of the weakly metastatic hormone-dependent cells of the LNCaP line, but this effect was usually small in magnitude and under many conditions, transient. Only DHA and AA could suppress this cell line’s viability, and then only at the highest concentrations. At concentrations 50 μM and above, PUFAs except LA suppressed LNCaP’s hormone-independent derivative C4-2 in a progressive concentration- and time-dependent manner. LA achieved only a slight and transient suppression of C4-2 at the highest concentration tested and, along with ALA, elicited some slight and transient stimulation of these cells’ growth at low concentrations.

The bird’s-eye view of this somewhat complicated picture appears to be, firstly, that the hormone-independent cell lines (PC3 and C4-2) responded to the tested PUFAs with a marked loss of viability. Secondly, little difference was found between *n*–3 and *n*–6 PUFAs. And by contrast, the hormone-dependent LNCaP, even though it is the precursor line of C4-2, was comparatively unaffected.

The new results extend the recent in vitro data on the sensitivity of prostate cancer cell lines to PUFA in the cell viability assay [23, 24] to incorporate the hormone-dependent line LNCaP and its hormone-independent derivative C4-2. Additionally, our experiments confirm that although the action of BSA-conjugated AA may not be detectable up to 24 h and 100 μM [24], at longer exposures it has a strong inhibitory effect on hormone-dependent prostate cancer cells, similar to the one that is developing more rapidly with unbound AA [23]. At the same time, the new dataset confirms that PUFA can be stimulatory to the prostate cancer cell proliferation under certain conditions–especially at the lowest concentrations, as was seen previously [21].

The negative effect of DHA on cell viability in our experiments can be explained mechanistically in the light of the recent work of Sun et al. on other hormone-dependent prostate cancer cells. The cited work [24] has shown a dynamic reprogramming of transcription indicative of upregulation of the apoptotic pathways and proapoptotic signaling. Considering the evidence for instances of wide interconversion, or metabolic cross-talk effects among the same set of PUFAs, which was obtained in the supplementation experiments by Meng et al. [23], not only the DHA precursors in the *n*–3 pathway but conceivably also the other PUFAs tested here might exert their effect through some of the same apoptotic mechanisms.

At the same time, it is likely that proapoptotic effects of AA may be primarily responsible for the action of the PUFAs in the *n*–6 pathway. It has been shown in retinoblastoma cells that administration of AA can lead to apoptosis, including activation of caspase 3 and cleavage of lamin B [36]. Similar results were obtained in hepatoma cells, where release of AA from phospholipids, downstream of an intracellular calcium concentration rise, mediated apoptosis by triggering the mitochondrial pathway [37]. Furthermore, in colon cancer cells, exogenous AA causes apoptosis, while accumulation of unesterified AA mediates the apoptosis under the conditions of COX-2 inhibition [38]. A similar mechanism has been uncovered in a lymphoma cell line [39]. The proapoptotic effect of 5-lipoxygenase inhibition in prostate cancer cells [22] may have similar underpinnings.

The new results are compatible with the mouse model [14] whereby *n*–3 PUFAs were found to be beneficial, while the different effect of *n*–6 PUFAs that was seen in vivo can, hypothetically, be attributable to the continued androgen stimulation in the mouse model, unlike in the experiments presented here. This is an interesting possibility to be investigated in appropriate experimental conditions in vitro. Alternatively, effects other than those exerted on the viability of the epithelial cells of the tumor (for example, inflammation-mediated ones) may be induced under the action of *n*–6 PUFAs in vivo. These possibilities could also account for the epidemiological observation of the importance for *n–*3 to *n*–6 ratio for development of high-grade prostate cancer [13].Extrapolating tentatively from these in vitro data, the hormone-dependent cells of patients undergoing hormone ablation therapy may be largely unaffected by PUFAs (including the nutritionally essential ones), whether *n*–3 or *n*–6. However, cells of the subpopulations that may at the same time be acquiring the more aggressive and hormone-independent phenotype are predicted to be suppressed by the same fatty acids. It remains to be determined if these results hold in vivo, and how the effects on the viability of cancerous cells of epithelial origin may be compounded by any additional effects mediated by the stroma and tumor microenvironment.
